# Oral Health-related Quality of Life of Children with Special Health Care Needs in Riyadh: A Cross-sectional Study

**DOI:** 10.3290/j.ohpd.b5573939

**Published:** 2024-07-23

**Authors:** Abrar Tounsi, AlBandary AlJameel, Maryam AlKathiri, Reem AlAhmari, Sarah Bin Sultan

**Affiliations:** a Assistant Professor, Department of Periodontics and Community Dentistry, College of Dentistry, King Saud University, Riyadh, Saudi Arabia. Idea, design, wrote the manuscript, proofread the manuscript, consulted on and performed statistical evaluation, contributed substantially to discussion.; b Associate Professor, Department of Periodontics and Community Dentistry, College of Dentistry, King Saud University, Riyadh, Saudi Arabia. Design, proofread the manuscript, consulted on and performed statistical evaluation.; c Dentist, College of Dentistry, King Saud University, Riyadh, Saudi Arabia. Idea, design, performed the experiments in partial fulfillment of requirements for a degree, wrote the manuscript.

**Keywords:** dental care for children, health-related quality of life, oral health, perception

## Abstract

**Purpose:**

To assess children’s OHRQoL and associated factors among a sample of children with special needs in Riyadh, Saudi Arabia.

**Materials and Methods:**

A sample of 6- to 12-year-old children was obtained using convenience sampling from rehabilitation centers. Data were collected through a questionnaire and dental examination. The questionnaire included items related to the children’s and their families’ characteristics, oral health-related quality of life scales (Parental-Caregivers Perceptions Questionnaire [P-CPQ] and Family Impact Scale [FIS]), perceived health status, and dental care utilisation. Clinical examination was performed by a trained and calibrated dentist. Descriptive and inferential data analyses were also performed using SPSS.

**Results:**

The mean P-CPQ was 1.10 ± 0.74, and the mean FIS was 1.39 ± 0.88. There was a statistically significant correlation between P-CPQ and caries (r = 0.36, p = 0.02). After controlling for confounders, caries was associated with poor P-CPQ (B = 0.06, p = 0.024). Compared to low-income families, higher-income families had better P-CPQ (4000-8000 SAR: B = -1.36, p = 0.001).

**Conclusion:**

Poor oral health-related quality of life in Saudi children is associated with caries and low income. Preventive measures addressing social determinants are vital to control caries and promote oral health in children with special healthcare needs.

The United States Department of Health and Human Services defines Children with Special HealthCare Needs (CSHCN) as those “who have or are at risk for developing a chronic physical, developmental, behavioral, or emotional condition and who also require health and related services of a type or amount beyond that required by children generally.”^10^ Disability is a multifaceted, complicated, influential, dynamic, and multidimensional difficulty that can severely limit an individual’s ability to engage in key life activities, as well as their ability to integrate or reintegrate into society.^22^

According to the World Health Organization reports, almost 15% of the world’s population lives with certain type of disability, of whom 2% to 4% experience substantial difficulties in functioning.^22^ In Saudi Arabia, very limited research has been conducted on the prevalence and incidence of disability, and most of existing literature is on children with disabilities.^22^ Around one million people in Saudi Arabia alone have one or more disabilities, which emphasises the need for particular care for this population.^4^

Individuals with Special HealthCare Needs (SHCN) are one of society’s most marginalised groups. Lack of care prevents them from receiving many of their rights, particularly in healthcare.^4^ In several previous studies,^4^^,^^23^^,^^24^ dental care has been recognised as one of the most common unmet healthcare needs among people with SHCN. While a large percentage of the global population still lacks access to dental care, people with SHCN are at and even higher risk of not receiving the dental treatment they need to maintain their oral health and manage dental diseases.^4^

The effects of dental disorders on overall health and function appear to be higher in people with disabilities than in people without disabilities.^14^ Due to a combination of reasons, such as compromised immunity, financial difficulties for parents, and resistance to dental treatment, CSHCN are at a higher risk for caries and other oral disorders. Furthermore, oral hygiene at home, particularly toothbrushing, can be challenging for CSHCN. Some children have sensory problems that makes using toothbrushes or fluoridated toothpaste challenging, while others lack the motor skills to brush their teeth on their own.^10^

Because of the scarcity of resources and limited access to dental health-care facilities, preventing oral disease in people with disabilities is crucial.^2^ Oral health is integral to children’s overall health and well-being. It is generally believed that good oral health can improve overall health, self-esteem, social integration, and quality of life (QoL). When oral healthcare does not meet the needs of individuals, it can have a negative impact on their overall health and well-being, leading to lower QoL. Poor oral health status can result in pain, sleep disturbances, decreased self-esteem, discomfort, and an unsatisfactory diet.^24^

The global burden of oral illnesses, such as caries, periodontal disease, and tooth loss tend to be high. Previous research has found that demographic and socioeconomic characteristics, such as education, occupation, income, and healthcare utilisation are associated with oral health problems.^13^ According to a study in Saudi Arabia, 46.2% of people with SHCN have difficulty getting dental care, and 84.7% are only seen at the dental clinic for emergency treatment.^4^ Several obstacles have been related to the limited access to dental care among individuals with SHCN, such as low income, inadequate parental education, and lack of dental insurance coverage.^4^

Determining the oral health-related quality of life (OHRQoL) is essential to better plan oral health prevention and treatment for CSHCN. In Saudi Arabia, the existing literature about oral health and its effect on the quality of life among CSHCN is limited.^4^^,^^6^^,^^22^ Therefore, the aim of this study was to explore children’s oral health-related quality of life (COHRQoL) in a sample of CSHCN, factors associated with COHRQoL, including its association with caries and gingival health.

## MATERIALS AND METHODS

This cross-sectional study was conducted with CSHCN and their caregivers in Riyadh city. The inclusion criteria for this study were: children from 6 to 12 years old, with at least one type of disability and enrolled in one of the services provided in the selected rehabilitation centers. Exclusion criteria were: uncooperative children, and caregivers who refused to sign the consent.

There are about 60 rehabilitation centers for children around Riyadh, ten of which were randomly selected from different areas of the city. Three out of the ten selected rehabilitation centers showed interest and willingness to participate in our project during the pandemic after verbal communication. Families of all children registered in these three centers were invited to participate, fill out the printed questionnaire, and to consent to their child’s oral examination. The eligibility for inclusion of children who returned the questionnaire was assessed using the criteria defined earlier.

The study proposal was ethically approved by King Saud University Institutional Review Board (E-21-6310). All caregivers received the informed consent form, which included a brief description of the study and its objectives. The complete choice to be enrolled and fill out the survey was completely voluntary.

### Data Collection/Data Source

The study consisted of two parts: a questionnaire and dental examination. The paper-based informed consent and questionnaire in Arabic language were sent to caregivers prior to the dental examination. Once the survey was completed and the consent signed, the dental examination was carried out. Only children with complete surveys and dental examination were included in the present study.

The questionnaire was distributed through rehabilitation centers to be filled between September 2021 and March 2022. The questionnaire was divided into child and family characteristics, dental care utilisation, and COHRQoL.^12^^,^^15^ Child’s characteristics included age, gender, number of associated disabilities, duration of disability, comorbidities, history of hospital admission, and perceived general health and oral health. Family characteristics involved respondent’s relationship to the child, education, family monthly income, employment status, caregivers’ general and oral health, having an assistant and father in the family, and number of siblings. Child’s dental care utilisation included the time of last dental visit, frequency of dental visits, and difficulties in getting dental care.

The second part contained two scales of COHRQoL: the Arabic version of Parental-Caregivers Perceptions Questionnaire (P-CPQ-8)^5^ and Family Impact Scale (FIS-8).^16^ P-CPQ-8 consists of eight questions measuring oral symptoms (2 items), functional limitations (2 items), emotional well-being (2 items) and 2 items for social well-being. FIS-8 comprises eight items addressing family activity (4 items), parental emotions (2 items), and family conflict (2 items). The questions in both scales referred to the frequency of events occurring during the previous 3 months. A five-point Likert-like scale was used with the following options of response: “never” (score 0), “once or twice” (1), “sometimes” (2), “often” (3) and “nearly every day” (4). The overall score was computed by summing the scores for all items in each scale and ranged from 0 to 32. A higher scale score represents worse or poor COHRQoL.

A general dentist performed the examination after being trained and calibrated to evaluate caries in primary and permanent dentitions and gingival health. The examiner was trained by a consultant in paediatric dentistry for 4 h per day for two days, following World Health Organization diagnostic criteria (2013). Duplicate examinations were conducted after three weeks on ten participants to assess intra-examiner reliability before starting the data collection. Intra-class correlation for caries was 0.96 (95%CI = 0.87, 0.99), and Kappa statistics for MGI was 0.68. The dental examination was carried out at rehabilitation centers on a regular chair in a private room under normal daylight conditions and using disposable instruments (gowns, gloves, mouth mirror, probe, and gauze). Each participant was examined individually in a private room with the attendance of his/her therapist to facilitate communication with the child through the examination process. No radiographic assessment was done. Caries was measured using the decayed, missing and filled teeth for permanent dentition (DMFT),^19^ the decayed, extracted and filled teeth in primary teeth (deft) and gingival health using the Modified Gingival Index (MGI).

For caries, the number of permanent and primary teeth that were either decayed, missing due to caries, or filled was recorded following a specific protocol. The decayed status was recorded when a lesion presented on the crown that had a visible cavity, undermined enamel, or a detectably softened floor or wall within the pit or fissure, or on a soft tooth surface. There was no distinction between primary and secondary decay of the tooth, and the same code was applied whether the carious lesions were in contact with the restoration or not. For missing teeth, it was recorded in the presence of missing tooth because of tooth decay. This implies permanent or primary teeth extraction due solely to caries. This score was not used for congenitally absent or un-erupted permanent teeth. Filled lesions were considered in the case of filled crown, with no tooth decay.

The Modified Gingival Index (MGI) uses a visual scale to assess gingival health. The MGI relies on a visual assessment of gingival changes to measure the severity of inflammation. The following scores were adopted: “0” in the absence of inflammation, “1” when there was mild inflammation or slight changes in colour and texture but not in all portions of gingival marginal or papillary, “2” in case of mild inflammation, such as the preceding criteria, in all portions of gingival marginal or papillary, “3” for moderate, bright surface inflammation, erythema, edema and/or hypertrophy of gingival marginal or papillary, and “4” for severe inflammation presented as erythema, oedema and/or marginal gingival hypertrophy of the unit or spontaneous bleeding, papillary, congestion or ulceration.

### Statistical Analysis

Data analyses were performed using SPSS version 26.0 (IBM; Armonk, NY, USA). Descriptive statistics were presented as counts and percentages for categorical variables, and as means with standard deviation for numerical variables. Intra-examiner reliability was assessed using intraclass correlation for continuous outcomes and kappa statistics for categorical outcomes. Bivariate analyses were conducted using the t-test and one-way ANOVA to assess the association for each of P-CPQ and FIS with child and family characteristics and child dental care utilisation, while outcomes association with MGI was assessed using the chi-squared test. Pearson’s correlation was used to assess the relationship of P-CPQ and FIS with caries. Linear regression was further conducted to assess the influence of factors, that were related to P-CPQ at bivariate analyses, in a multivariable environment and was presented as beta coefficients, 95% confidence interval (CI), and p-value. For linear regression model, the objective measure (caries: DMFT and deft) of oral health were preferably used over the subjective measure (child’s perceived oral health). Statistical significance was defined as p<0.05.

## RESULTS

A total of 40 children with various types of disabilities participated in the present study, where 50% had developmental disabilities (mainly autism and ADHD), 17.5% had Down’s syndrome, 10% had intellectual disabilities, and 5% had motor disabilities. The sample distributions of child and family characteristics are presented in Table 1. About 47.5% of the sample were boys and 52.5% were girls, with a mean age of 9.12 ± 2.29. More than half of the children had a single type of disability (n = 23, 57.5%), while the remaining had more than one disability. Most of the children had their disability since birth (n = 25, 62.5%) and no other chronic diseases (n = 37, 92.5%).

**Table 1:** Distribution of child and family characteristics among the study sample

Variable	Categories	Freq (%)	Variable	Categories	Freq (%)
Gender	Girls	21 (52.5)	Caregiver’s relationship	Mother	38 (95.0)
	Boys	19 (47.5)		Others	2 (5.0)
Number of associated disabilities	None	23 (57.5)	Caregiver’s education	Primary school or less	4 (10.0)
	One	8 (20.0)		Intermediate	4 (10.0)
	Two	6 (15.0)		High school	4 (10.0)
	Three or more	3 (7.5)		Bachelor’s degree	24 (60.0)
Duration of disability	Since born	25 (62.5)		Higher education	3 (7.5)
	Less than 1 year	3 (7.5)	Family monthly income	Less than 4,000 SAR	5 (12.5)
	1 – 3 years	9 (22.5)		4,000 – 8,000 SAR	7 (17.5)
	3 years or more	3 (7.5)		8,000 – 12,000 SAR	9 (22.5)
Comorbidity	Yes	3 (7.5)		More than 12,000 SAR	19 (47.5)
	No	37 (92.5)	Employment status	None	4 (10.0)
Admission	Yes	6 (15.0)		One of them	21 (52.5)
	No	34 (85.0)		Both	15 (37.5)
Child’s perceived general health	Good	24 (60.0)	Caregiver’s general health	Good	17 (42.5)
	Excellent	16 (40.0)		Excellent	23 (57.5)
Child’s perceived oral health	Bad	7 (17.5)	Caregiver’s oral health	Bad	4 (10.0)
	Good	26 (65.0)		Good	23 (57.5)
	Excellent	7 (17.5)		Excellent	13 (32.5)
Frequency of toothbrushing	None	4 (10.0)	Assistant presence	Yes	19 (47.5)
	Once a day	23 (57.5)		No	21 (52.5)
	Twice or more	13 (32.5)	Father presence	Yes	37 (92.5)
Any dental visit	No	12 (30.0)		No	3 (7.5)
	Yes	28 (70.0)	Number of siblings	None	3 (7.5)
Last dental visit	6 months	12 (30.0)		One sibling	11(27.5)
	Within one year	4 (10.0)		More than one	26 (65.0)
	More than a year	12 (30.0)			
Frequency of dental visit	None	12 (30.0)			
	Emergency	20 (50.0)			
	Every 6 months	4 (10.0)			
	Every 12 months	4 (10.0)			
Any difficulties in getting dental care	Yes	20 (50.0)			
	No	12 (30.0)			
	Did not seek dental care	8 (20.0)			

Most children had a father (92.5%), and two-thirds of children had more than one sibling (65%). Almost half of the families (47.5%) had an assistant. About 67.5% of caregivers had a bachelor’s degree or higher. Only 10% of parents were not employed, and 47.5% of families had a monthly income of 12,000 SAR or more.

Most of the children had good or excellent oral health (82.5%), while 17.5% had poor oral health. Almost two-thirds (n = 27) of the children had visited a dentist before, and 40% (n = 16) had had their last dental visit within the preceding year or less. Also, half of the children visited the dental clinic only for an emergency. Approximately 50% of children had difficulty accessing dental care.

### Distribution of COHRQoL 

As shown in Table 2, the mean P-CPQ for our sample was 1.10 ± 0.74 and mean FIS was 1.39 ± 0.88. No association was found between COHRQoL domains and child’s characteristics such as gender, number of disabilities, comorbidities, and child’s general health. However, there was a statistically significant association between child’s perceived oral health and P-CPQ (p = 0.03). Children with poor perceived oral health had a statistically significantly poor P-CPQ (1.71 ± 0.46) compared to good perceived oral health (0.92 ± 0.70).

**Table 2:** Comparing child oral health-related quality of life across child characteristics

		Mean P-CPQ ± SD	p-value	Mean FIS ± SD	p-value
Total		1.10 ± 0.74		1.39 ± 0.88	
Gender	Girls	1.30 ± 0.67	0.07	1.63 ± 0.82	0.07
	Boys	0.88 ± 0.76		1.13 ± 0.88	
Number of associated disabilities	None	1.17 ± 0.85	0.93	1.44 ± 0.99	0.28
	One	1.05 ± 0.62		1.28 ± 0.76	
	Two	1.02 ± 0.61		1.77 ± 0.17	
	Three or more	0.92 ± 0.44		0.58 ± 0.80	
Duration of disability	Since born	1.22 ± 0.77	0.39	1.54 ± 0.85	0.47
	Less than 1 year	0.58 ± 0.07		0.75 ± 0.90	
	1 – 3 years	0.89 ± 0.78		1.22 ± 0.97	
	3 years or more	1.33 ± 0.38		1.38 ± 0.88	
Comorbidity	Yes	1.13 ± 0.54	0.96	1.21 ± 0.85	0.71
	No	1.10 ± 0.76		1.41 ± 0.89	
Admission	Yes	1.27 ± 0.62	0.55	1.42 ± 0.81	0.95
	No	1.07 ± 0.76		1.39 ± 0.90	
Child’s perceived general health	Good	1.19 ± 0.79	0.38	1.45 ± 0.80	0.64
	Excellent	0.98 ± 0.65		1.31 ± 1.00	
Child’s perceived oral health	Bad	1.71 ± 0.46*	0.03	1.95 ± 0.47	0.18
	Good	0.92 ± 0.70*		1.30 ± 0.89	
	Excellent	1.18 ± 0.81		1.20 ± 1.01	
Frequency of toothbrushing	None	0.50 ± 0.37	0.21	0.66 ± 0.43	0.20
	Once a day	1.13 ± 0.73		1.51 ± 0.82	
	Twice or more	1.24 ± 0.78		1.42 ± 1.01	

SD = standard deviation, P-CPQ = parental-caregiver perceptions questionnaire, FIS = family impact scale. The t-test was used to compare the mean of dichotomous variables and one-way ANOVA to compare variables of more than two categories. *Statistically different at p<0.05.

The association of family’s characteristics with COHRQoL was assessed in Table 3. Caregiver’s education, employment status, general and oral health, and number of siblings in the family did not statistically significantly influence P-CPQ and FIS. Family income was the only family character statistically significantly associated with P-CPQ (p = 0.007). The mean P-CPQ among families of monthly income less than 4000 SAR (2.00 ± 0.70) was statistically significantly higher than the mean P-CPQ for families of monthly income more than 12,000 SAR (1.04 ± 0.68).

**Table 3:** Comparing child oral health-related quality of life across family characteristics

		Mean P-CPQ ± SD	p-value	Mean FIS ± SD	p-value
Caregiver’s relation	Mother	1.11 ± 0.72	0.84	1.40 ± 0.86	0.74
	Others	1.00 ± 1.41		1.19 ± 1.68	
Caregiver’s education	Primary school or less	0.94 ± 0.59	0.88	1.40 ± 0.62	0.19
	Intermediate	1.22 ± 1.15		0.56 ± 0.97	
	High school	1.34 ± 0.84		1.47 ± 0.69	
	Bachelor’s degree	1.10 ± 0.75		1.41 ± 0.89	
	Higher education	0.79 ± 0.51		2.21 ± 0.79	
Family monthly income	Less than 4,000 SAR	2.00 ± 0.70*, **	0.007	1.68 ± 1.09	0.79
	4,000 - 8,000 SAR	0.59 ± 0.58*		1.14 ± 0.84	
	8,000 -12,000 SAR	1.14 ± 0.60		1.43 ± 1.02	
	More than 12,000 SAR	1.04 ± 0.68**		1.39 ± 0.82	
Employment status	None	1.78 ± 0.50	0.15	1.00 ± 0.85	0.46
	One of them	1.03 ± 0.81		1.33 ± 0.99	
	Both	1.03 ± 0.62		1.58 ± 0.72	
Caregiver’s general health	Good	1.10 ± 0.88	0.99	1.38 ± 0.92	0.91
	Excellent	1.10 ± 0.63		1.41 ± 0.86	
Caregiver’s oral health	Bad	1.41 ± 0.61	0.66	1.56 ± 0.77	0.69
	Good	1.04 ± 0.83		1.29 ± 0.86	
	Excellent	1.13 ± 0.61		1.53 ± 0.98	
Assistant presence	Yes	1.07 ± 0.63	0.80	1.47 ± 0.91	0.59
	No	1.13 ± 0.84		1.32 ± 0.86	
Father presence	Yes	1.04 ± 0.68	0.07	1.35 ± 0.85	0.29
	No	1.83 ± 1.19		1.92 ± 1.28	
Number of siblings	None	0.71 ± 0.36	0.36	1.79 ± 0.07	0.16
	One sibling	0.93 ± 0.63		0.98 ± 0.78	
	More than one	1.22 ± 0.79		1.52 ± 0.92	

SD = standard deviation, P-CPQ = parental-caregiver perceptions questionnaire, FIS = family impact scale. The t-test was used to compare the mean of dichotomous variables and one-way ANOVA to compare variables of more than two categories. Similar symbols are statistically significantly different at p<0.05.

Table 4 shows the distribution of COHRQoL by dental care utilisation. Even though children who visited dentists only for emergencies (P-CPQ: 1.33 ± 0.77, FIS: 1.60 ± 0.91) and who had difficulties in getting dental care (P-CPQ: 1.23 ± 0.67) were more likely to have poor COHRQoL, there was no statistically significant association between COHRQoL and the pattern of seeking dental care.

**Table 4:** Comparison of child oral health-related quality of life by child’s dental care utilisation

		Mean P-CPQ ± SD	p-value	Mean FIS ± SD	p-value
Dental visit	No	1.07 ± 0.96	0.86	1.11 ± 1.07	0.15
	Yes	1.12 ± 0.62		1.53 ± 0.75	
Last dental visit	6 months	1.03 ± 0.59	0.46	1.69 ± 0.33	0.37
	Within one year	1.56 ± 0.78		1.06 ± 1.04	
	More than a year	1.13 ± 0.81		1.63 ± 0.97	
Frequency of dental visit	None	0.82 ± 0.73	0.26	0.94 ± 0.95	0.19
	Emergency	1.33 ± 0.77		1.60 ± 0.91	
	Every 6 months	0.84 ± 0.67		1.50 ± 0.35	
	Every 12 months	1.09 ± 0.47		1.63 ± 0.43	
Any difficulties in getting dental care	Yes	1.23 ± 0.67	0.38	1.35 ± 0.81	0.95
	No	0.85 ± 0.73		1.43 ± 1.00	
	Did not seek dental care	1.16 ± 0.91		1.45 ± 0.97	

The t-test was used to compare the mean of dichotomous variables and one-way ANOVA to compare variables of more than two categories. n = frequency, SD=standard deviation, P-CPQ=parental-caregiver perceptions questionnaire, FIS=family impact scale.

### Association with Oral Health Indicators (Objective Measures)

There was a statistically significant correlation between P-CPQ and caries (r = 0.36, p = 0.02). On the other hand, FIS was not statistically significantly correlated with caries (r= 0.13, p = 0.43). In regard to MGI, neither P-CPQ nor FIS were statistically significantly associated with MGI (p = 0.55 and 0.49, respectively).

### Multiple Linear Regression for P-CPQ

Factors that were statistically significantly associated with P-CPQ in bivariate analysis were further assessed in a multivariable analysis, as shown in Table 5. Caries experience was associated with poor P-CPQ (B = 0.06, 95%CI = 0.01- 0.11, p = 0.024) after controlling for confounders. Compared to low-income families, higher income families had better P-CPQ (4000-8000 SAR: B = -1.36, 95%CI = -2.09- -0.63, p = 0.001).

**Table 5:** Multiple linear regression for P-CPQ

Factor	Levels	B coefficient	95% confidence interval		p-value
			Lower	Upper	
Dental caries (DMFT and deft)		0.06	0.01	0.11	0.024
Family income	Less than 4,000 SAR	Reference			
	4,000 – 8,000 SAR	-1.36	-2.09	-0.63	0.001
	8,000 – 12,000 SAR	-0.79	-1.49	-0.09	0.026
	More than 12,000 SAR	-0.81	-1.45	-0.17	0.014

## DISCUSSION

The life of children with special healthcare needs and their families is undoubtedly full of challenges due to their health condition. As oral health is an integral part of overall health, our study aimed to assess children’s oral health-related quality of life, factors related to it, and its relationship with oral health indicators among a sample of CSHCN. To our knowledge, few studies have assessed the COHRQoL among CSHCN.^11^^,^^17^ Shedding the light on this aspect would help in understanding areas in need of improvements for CSHCN in Saudi Arabia.

The present study revealed that a high percentage of our sample of CSHCN had good or excellent perceived oral health (82.5%), while 17.5% had perceived poor oral health. In contrast, most caregivers acknowledged that it is difficult and challenging to care for CSHCN oral health compared to their healthy peers among a sample of caregivers of SCHCN in Al-Kharj, Saudi Arabia.^22^ Multiple factors – including their educational level and income – could shape how caregivers perceive their children’s oral health.^20^

Multiple previous studies have addressed the deleterious effect of caries on the OHRQoL in individuals with or without disabilities.^3^^,^^9^^,^^21^ Our study indicated that there was a statistically significant association between both subjective and objective measures of child’s oral health and COHRQoL. Subjective measure involved child’s perceived oral health status, while objective measures included clinical examination of caries experience and gingival health. Children with poor perceived oral health had a statistically significantly poor P-CPQ (1.71 ± 0.46) compared to good perceived oral health (0.92 ± 0.70).

In addition, there was a positive correlation between caries and P-CPQ (r=0.36, p=0.02). Although this association is supported by a former investigation^3^ in which caries experience had a gradient association with COHRQoL measured by Early Childhood Oral Health Impact Scale (ECHOIS), our findings did not agree with those of an earlier study^18^ that assessed the association of caries with P-CPQ in special healthcare needs children. In studies by Nqcobo et al,^17^^,^^18^ there was no association between caries and P-CPQ; as those authors discussed, this variation could be attributed to the low prevalence of caries among the sample, which was selected from an outreach site for CSHCN, in which caregivers received frequent oral health education at the discussion groups.

Although our study detected no effect of caries on FIS, the impact of severity of caries on families of children with special needs had been documented in a Brazilian study,^8^ in which the impact was demonstrated specifically in the parental emotions domain. Despite the fact that no statistically significant association was detected, the gradient influence of caries severity on FIS was shown in a sample of children with cerebral palsy.^1^

Among family characteristics, family income was statistically significantly associated with P-CPQ. Low-income families had statistically significantly poorer COHRQoL compared to high-income families. A similar association was revealed in a study by Alvarenga et al,^7^ in which low income was associated with negative impact on the “psychological disability” domain of the Oral Health Impact Profile (OHIP-14) in a sample of children with cerebral palsy. This relationship emphasises the role of economic status as a social determinant of health, and the necessity of addressing this factor to improve the oral health of our communities.

In the present study, half of the children visited a dental clinic only for emergencies, and approximately 50% reported difficulty in accessing dental care. Comparably, the majority (85%) of CSHCN visited the dentist only for emergencies in a previous study in Saudi Arabia, indicating that oral health diseases and oral health become a concern among this population only when symptoms such as pain arise.^6^ The difficulty this population faces in receiving dental care in Saudi Arabia has been previously discussed by Al-Shehri,^6^ and could also be a contributing factor to the increased tendency toward emergency-oriented dental visits. Fear, cost, uncooperative behaviour, etc. are among the most prevalent barriers for CSHCN to accessing dental care regularly.

Our study furnished valuable information about OHRQoL among CSHCN in Saudi Arabia using a validated P-CPQ and FIS scales, and included their correlation with both subjective and objective oral health measures. However, study limitations must be considered in the interpretation of the results of this study. Firstly, caution should be taken when generalising the findings of the present study due to its limited sample size and the difficulty of collecting data during the pandemic. Another limitation of our study was its cross-sectional nature where causal inferences cannot be made. In addition, it is essential to mention that using WHO criteria for caries detection could have led to underestimating the true level of caries among this population. Future studies should direct their efforts toward addressing the variation existed in OHRQoL in the literature related to CSHCN. In addition, the effect of preventive measures and social determinants approaches on OHRQoL of CSHCN should be explored in the future.

## CONCLUSION

The oral health of CSHCN impacts the quality of their and their families’ lives, and this impact is more prominent among families from low-income groups. Dental care is an integral component of general health; therefore, it should always be included in the comprehensive medical care for CSHCN. An appropriate and targeted strategy is needed to address relevant socioeconomic and behavioural factors to improve OHRQoL. Public health measures should focus on the prevention of caries among CSHCN and the development of strategies to promote oral health in Saudi Arabia, especially among economically disadvantaged families.
